# Relationship of four vitamin D receptor gene polymorphisms with type 1 diabetes mellitus susceptibility in Kuwaiti children

**DOI:** 10.1186/s12887-019-1448-0

**Published:** 2019-03-07

**Authors:** Majedah A. Rasoul, Mohammad Z. Haider, Maria Al-Mahdi, Hessa Al-Kandari, Gursev S. Dhaunsi

**Affiliations:** 1 Department of Pediatrics, Faculty of Medicine, Kuwait University, P.O. Box 24923, Safat-13110 Jabriya, Kuwait; 2 Department of Pediatrics, Adan Hospital, Al-Adan, Kuwait; 3 Department of Pediatrics, Farwania Hospital, Farwania, Kuwait; 4 Family Medicine and Pediatric Unit, Dasman Diabetes Institute, Dasman, Kuwait; 5 Medical Laboratories, Mubarak Al-Kabeer Hospital, Jabriya, Kuwait

**Keywords:** Autoantibody, Genotype, Vitamin D receptor gene, Type 1 diabetes mellitus, Polymorphism, Kuwait

## Abstract

**Background:**

The incidence of type 1 diabetes mellitus (T1DM) in Kuwait is amongst the highest in the world. Vitamin D is considered to be involved in immune modulation and its deficiency contribute to autoimmune destruction of insulin producing beta cells in T1DM patients. Vitamin D has been shown to exert its effects via a nuclear vitamin D receptor (VDR) and therefore, *VDR* gene may be considered a candidate for T1DM susceptibility.

**Methods:**

The genotypes of four *VDR* gene polymorphisms were determined in 253 Kuwaiti Arab T1DM patients and 214 healthy controls by PCR-RFLP analysis. Serum concentrations of three autoantibodies i.e. ICA (Islet cell autoantibody), GADA (Glutamic acid decarboxylase) and INS (Insulin autoantibody) were determined by radio-immunoassays.

**Results:**

Statistically significant differences were detected between the genotypes of two *VDR* gene polymorphisms (*Fok*I, C > T, rs10735810 and *Taq*I, C > T, rs731236) between T1DM patients and controls (*P* < 0.0001). In both, the frequency of variant alleles was considerably high in T1DM than in the controls. In contrast, the *VDR* gene *Apa*I (G > T, rs7975232) and *Bsm*I (A > G, rs1544410) polymorphisms did not show association with T1DM. The homozygous variant genotypes of *Fok*I, *Apa*I and *Taq*I polymorphisms show significant differences between various age-of-onset subgroups while no such association was detected in the case of *Bsm*I polymorphism. Significant differences were also noted between heterozygous genotypes of all four polymorphisms especially between 4-6y and > 6y age-of-onset subgroups of T1DM patients. Three autoantibodies, ICA (Islet cell), GADA (glutamate decarboxylase) and INS (insulin) were positively associated to, varying degrees, with T1DM in Kuwaiti Arabs harboring different *VDR* gene polymorphism genotypes.

**Conclusions:**

Our results demonstrate a significant effect of two *VDR* gene polymorphisms (*Fok*I and *Taq*I) and three autoantibodies on genetic susceptibility of T1DM in Kuwaiti Arabs along with other factors.

## Background

Type 1 diabetes mellitus (T1DM) is a chronic multifactorial disease that is considered to be caused by selective autoimmune destruction of the pancreatic beta-cells which makes the affected individual dependent on exogenous insulin. Recent epidemiological data from Kuwait [1] showed a considerable increase in the incidence of T1DM (40.9 per 100,000) compared to 3.96 per 100,000 individuals reported in the 1980–81 [2]. This alarming increase in the T1DM incidence rate has moved Kuwait to the list of high-incidence countries. A wide variation in the incidence of T1DM has been reported across the globe with highest incidence found in Scandinavian countries and lowest in China and Japan [3]. It has been suggested that environmental factors, nutritional pattern, life style changes along with genetic susceptibility may be responsible for this wide variation and rapid increase in the incidence of T1DM in different world populations.

Several past reports have demonstrated that serum vitamin D deficiency is wide spread around the world [4–8]. It has been shown that the vitamin D deficiency is involved in induction of autoimmune destruction of beta-cells and onset of T1DM through the loss of immunomodulation (vitamin D favors Th2 response and protects from further beta-cell destruction [9, 10]). There is also evidence which show that the risk of T1DM from vitamin D deficiency has increased over the years [11, 12]. It has been reported that vitamin D activates human macrophages, antigen presenting cell maturation, has a negative effect on dendritic cell differentiation and is involved in the regulation of cytokine production by interacting with immune cells and may therefore be considered an immune system modifier [13]. Also, it has been shown that the administration of vitamin D has a protective effect against T1DM in non-obese diabetic (NOD) mice [14]. Previous reports have shown that the dietary intake of vitamin D in early childhood decreased the risk of T1DM [15, 16]. Also, the maternal vitamin D supplementation during pregnancy resulted in protection from appearance of islet cell autoantibodies in newborn babies [17]. Vitamin D exert its actions via a nuclear vitamin D receptor (VDR) therefore the *VDR* gene can be considered a candidate/susceptibility gene for T1DM [18]. The *VDR* gene contain eight protein-encoding exons (exons 2–9) and six untranslated exons (exons 1a-1f), and is located on chromosome 12 [19]. The VDR belongs to the steroid receptor super-family and is expressed in many cell types which include lymphocytes, macrophages, and pancreatic cells [20]. A number of single nucleotide polymorphisms (SNPs) have been reported in the *VDR* gene; these include *Fok*I, (rs10735810), *Apa*I (rs7975232), *Taq*I (rs731236) and *Bsm*I (rs1544410) [18]. In the case of *Fok*I, the presence of T creates an additional ATG initiation codon in exon 2, which result in VDR protein that is three amino acids longer. The *Apa*I and *Bsm*I polymorphisms are located in intron 8 while the silent *Taq*I polymorphism is present in intron 9 [18]. It has been reported that the *VDR* gene polymorphisms have been associated with altered gene expression or gene function [21]. These four *VDR* gene polymorphisms have been studied in several different populations (including India, Japan, Iran and Finland) for their association with T1DM with inconsistent results [22–25]. A study from South India showed that b-allele of the *VDR* gene, *Bsm*I polymorphism was associated with T1DM [22]. However, no association was detected between *VDR* gene polymorphisms and T1DM in patients from Finland [25]. Boraska et al. [26] reported an association of *VDR* gene *Tru9*I polymorphism and *Tru9*I-*Bsm*I haplotypes with T1DM in patients from South Croatia. In contrast, no significant association between three *VDR* gene polymorphisms (*Bsm*I, *Apa*I and *Taq*I) and T1DM was detected in patients from Chile [27]. The data from Sudan, showed an association of two *VDR* gene polymorphisms (*Bsm*I and *Taq*I) with T1DM [28]. In a study from Pakistan, *VDR* gene polymorphisms *Fok*I and *Apa*I were found to be associated with T1DM while no association was found with the *Taq*I polymorphism [29]. A positive association between *VDR* gene polymorphisms and T1DM has also been reported in Chinese Han population [30], in Taiwan [31], in Saudi Arabia [32], in Korea [33] and in Spain [34]. However, in contrast to these positive associations, a study from Denmark, reported no association between type 1 diabetes and genetic variation in vitamin D metabolism genes [35]. The relationship between inherited variation in vitamin D genes and diabetes has been addressed in recent reviews and meta-analyses [36–38]. In view of the divergent findings in different populations/ethnic groups, we determined the prevalence of four *VDR* gene polymorphisms in Kuwaiti Arab children and investigated their correlation with age-of-onset of the disease in order to evaluate their influence on susceptibility to T1DM in a completely different population/ethnic group. In addition, we also determined the serum concentrations of three autoantibodies in Kuwaiti children with T1DM and explored their pattern of association with different *VDR* gene polymorphism genotypes.

## Methods

This study had a ‘case-control’ design and included 253 newly diagnosed T1DM patients from three major hospitals (Adan, Farwania and Mubarak Al-Kabeer). A large sub-set of T1DM patients from this report had been included in our earlier publications [8, 39]. The diagnosis and inclusion criteria employed have been described earlier and were based on that recommended by the ISPAD protocol [40]. In order to investigate an association of *VDR* gene polymorphisms with age of onset of T1DM, the patient group was divided into three sub-groups on the basis of age-of-onset of T1DM; 0-4y, 4-6y and 6-14y as reported earlier [41]. The age-of-onset subgrouping was modified slightly (instead of 5–9 y used in [41], we chose the 4-6y range due to a very high incidence of T1DM cases in earlier age in Kuwaitis. The glycemic status of study subjects was determined by measuring the serum levels of HbA1c by high performance liquid chromatography (HPLC). In addition to the T1DM patients, 214 non-diabetic healthy controls were also studied. The controls were volunteers, they can be considered as convenience samples, and were all Kuwaiti Nationals of similar ethnicity to the T1DM patients. The mean age in the T1DM patients group was 8.5 years (± 5.5) compared to 8.9 years (± 5.2) in the controls (no statistically significant difference between the two groups). In T1DM patients there were 125 males while the control group had 110 males (*P* = 0.5). The T1DM patients group contained 128 females compared to 104 in the controls (*P* = 0.51).

The details of sample collection and processing have been described previously [8]. For determination of the *VDR* genotypes, the blood was anti-coagulated with EDTA and the total genomic DNA was isolated by a standard method [42].

### *VDR* gene *Fok*I polymorphism

The *Fok*I polymorphism, C > T (rs10735810), in *VDR* gene was identified by using a polymerase chain reaction-restriction fragment length polymorphism (PCR-RFLP) method described earlier [43]. A 265 bp PCR product was amplified by using the primers: Forward: 5′-AGCTGGCCCTGGCACTGACTCTGCTCT-3′ and Reverse: 5′-ATGGAAACACCTTGCTTCTTCTCCCTC-3 [43]. The PCR mixture contained 200 ng DNA template, 20 nmol of each primer, 2.0 mM MgCl_2_, 0.2 mM dNTPs and 1 .0U AmpliTaq DNA polymerase (Applied BioSystems). The PCR reactions were carried out at 94 °C for 5 min followed by 35 cycles of 94 °C for 30 s, 60 °C for 30 s and 72 °C for 30 s and an extension step of 72 °C for 7 min [43]. The products of PCR amplification were cleaved with 10 U of restriction enzyme *Fok*I (New England BioLabs) at 37 °C for 90 min. The cleavage products were analyzed by agarose gel electrophoresis (2%) and visualized by ethidium bromide staining under UV light. Alleles ‘F’ or ‘f’ were assigned on the basis of presence of 265 bp fragment (f-allele) and 196 and 69 bp products (F-allele) respectively. In heterozygous Ff individuals, products of 265, 196 and 69 bp were present.

### *VDR* gene *Apa*I and *Taq*I polymorphisms

The g.59979G > T (*Apa*I; rs7975232) and g.60058T > C (*Taq*I; rs731236) polymorphic sites of the *VDR* gene were analyzed by PCR-RFLP method [44, 45]. A 740-bp fragment was amplified using the primers; Forward: 5′-CAGAGCATGGACAGGGAGCAAG-3′ and Reverse: 5′-GCAACTCCTCATGGCTGAGGTCTCA-3′ [44]. The PCR mixture contained 100 ng DNA template, 20 nmol of each primer, 2.2 mM MgCl_2_, 0.2 mM dNTPs and 0 .8U AmpliTaq DNA polymerase (Applied BioSystems). The PCR conditions used were: 94 °C for 5 min; 30 cycles consisting of 1 min at 94 °C, 1 min at 60 °C and 90 s at 72 °C followed by an extension step for 10 min at 72 °C. The PCR product was digested with 5 U *Apa*I (New England BioLabs) at 25 °C overnight. The products of restriction enzyme cleavage were separated by 2% agarose gel electrophoresis and were visualized under UV light after staining with Ethidium bromide. In the presence of T-allele, there was no restriction enzyme cleavage site and a product of 740 bp was obtained. In subjects carrying ‘G-allele’, the cleavage products of 530- and 212-bp were detected.

The same PCR product was digested with restriction enzyme *Taq*I (New England BioLabs) at 65 °C for 75 min to detect the *Taq*I polymorphism [44]. The products of restriction enzyme digestion were analyzed on 2% agarose gels and visualized under UV following staining with Ethidium bromide. Allele ‘T’ was associated with the presence of 495 bp and 245 bp cleavage products while allele ‘C’ was assigned in the presence of 290, 245 and 210-bp fragments respectively [44, 45].

### *VDR* gene *Bsm*I polymorphism (A > G, rs1544410)

This polymorphism was determined according to a modification of the previously published PCR-RFLP method [44]. The PCR amplification was carried out by using the primers, Forward: 5′-AACCAGCGGGAAGAGGTCAAGGG-3′ and Reverse: 5′-CAACCAAGACTACAAGTACCGCGTCAGTGA-3′ [44]. The PCR mixture contained 100 ng DNA template, 20 nmol of each primer, 1.5 mM MgCl_2_, 0.2 mM dNTPs and 1.0 U AmpliTaq DNA polymerase and the amplification conditions used were: 95 °C for 5 min; 30 cycles of 94 °C for 1 min, 57 °C for 1 min, 72 °C for 1 min followed by 10 min at 72 °C. Using the above conditions and ingredients, PCR amplification resulted in 870 bp PCR product. The PCR amplification products were cleaved with restriction enzyme *Mva*I (New England BioLabs) at 37 °C for 75 min. The products of the restriction enzyme digestion were analyzed using 2% agarose gel electrophoresis and visualized by UV light following staining with Ethidium bromide. Allele ‘B’ was assigned based on the presence of 870 bp (cleavage site absent) and allele ‘b’ was associated with the presence of products of 460, 234 and 176 bp respectively [44].

The haplotypes were built by using a previously published method [18] and were described in the order *Fok*I/*Taq*I/*Apa*I/*Bsm*I. The comparisons were made in the haplotype frequency between T1DM patients and the controls.

### Detection of autoantibodies

The method used for detection of three autoantibodies (Islet Cell autoantibody, ICA; glutamic acid decarboxylase, GADA and Insulin, INS autoantibodies has been described in our earlier report [39]. For GADA and ICA autoantibodies, the cut off values used were 180 and 80 counts per minute/microliter of the sera respectively. The patients were divided into autoantibody-positive and autoantibody-negative sub-groups.

### Statistical analysis

The data was analyzed by using the Statistical Package for Social Sciences version 25 (SPSS, Chicago IL, USA). Genotype and allele frequencies in T1DM patients and controls were calculated by direct counting. The continuous variables in the T1DM patient and control groups were compared using the student t-test. The categorical variables were analyzed using the Chi-square and the Fisher’s Exact test as appropriate. Odds ratios (OR) were calculated with 95% confidence interval (CI). The data in the three age-of-onset subgroups were further analyzed by Chi-Square test and the Fisher’s Exact test. The *P*-values < 0.05 were considered as significant. In order to calculate the statistical significance in co-dominant and dominant models, the genotype frequency in homozygous FF subjects and frequency of ‘F’ allele of the *Fok*I polymorphism, homozygous TT and frequency of ‘T’ allele of the *Taq*I polymorphism, genotype TT and allele T of the *Apa*I polymorphism and genotype BB and allele B of the *Bsm*I polymorphisms were considered as reference (assumed to be associated with the least risk of T1DM). In the dominant model, genotype frequencies of the heterozygous Ff and homozygous ff (for *Fok*I polymorphism), heterozygous Tt and homozygous tt genotypes in the case of *Taq*I polymorphisms, heterozygous TG and homozygous GG in *Apa*I polymorphism and heterozygous Bb and homozygous bb for *Bsm*I polymorphism were pooled (i.e. T1DM patients who had at least one presumed ‘susceptibility’ allele). A possible limitation of the study may be that the presence of low numbers in some comparisons e.g. age-of-onset in T1DM patients and with autoantibody-positivity, may result in type-II errors. Hardy Weinberg equilibrium was tested in the genotype distributions by goodness of fit method using MSTAT software.

## Results

**Fig. 1:**
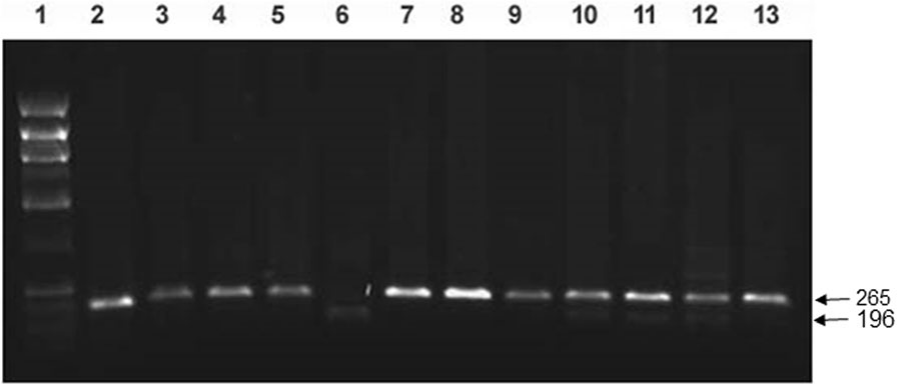
Detection of *VDR* gene *Fok*I polymorphism. PCR amplification of the genomic DNA was carried out and the products of amplification were cleaved with restriction enzyme *Fok*I (details given in Methods). Lane 1, phiX174 *Hae*III cut *M*_*r*_ markers; lane 2, uncleaved PCR product; lane 3–5,7–9 & 13, PCR products from subjects having ff genotype; lane 6, pattern from a subject with homozygous FF genotype; lanes 10–12, pattern from subjects with heterozygous Ff genotype. The numbers on the side are sizes (bp) of the characteristic bands resulting from PCR-RFLP analysis. The restriction enzyme cleavage products were analyzed on 2% agarose gel and visualized under UV light after staining with Ethidium bromide

Amongst the T1DM patient group, 193/253 (76%) had their HbA1c between 7 and 10% and in 60/253 (24%) it was > 10%. In all controls, the HbA1c level was below 6.5%. The mean serum vitamin D level in Kuwaiti T1DM patients was 12.88 ± 6.66 ng/ml and in the controls it was 15.02 ± 10.4 ng/ml. The general characteristics of the T1DM patients and controls have been described in a recent report from our group [39]. An example of the method employed for detecting the *VDR* gene *Fok*I polymorphism and the results obtained have been presented in Fig. 1. Statistically significant differences were detected in the case of homozygous ff genotype and the ‘f’ allele between T1DM patients and controls (OR 8.62; and 9.92 in co-dominant and dominant models respectively, Table 1). The frequency of variant ‘f’ allele was found to be considerably higher in T1DM patients while the ‘F’ allele was more prevalent in the controls (Table 1).

**Table 1:** Comparison of the genotype and allele frequency of *VDR* gene polymorphisms (*Fok*I*, and Taq*I) between Kuwaiti T1DM patients and controls

*VDR* gene polymorphism	Patients *N* (%)	Control N* (%)	OR (95% CI)**	*P*–value***
*Fok*I				
Co-dominant	N = 253	N = 214		
FF	45 (17.8)	146 (68.2)	1.00 (Reference)^a^	
Ff	30 (11.9)	1 (0.5)	97.3 (12.90–73.24)	< 0.0001
ff	178 (70.3)	67 (31.3)	8.62 (5.57–13.35)	< 0.0001
Dominant				
FF	45 (17.8)	146 (68.2)	1.00 (Reference)^a^	
Ff/ff (Ff + ff)	208 (82.2)	68 (31.8)	9.92 (6.44–15.28)	< 0.0001
Alleles	*N* = 506	*N* = 428		
F	120 (23.7)	293 (68.51)	1.00 (Reference)^a^	
f	386 (76.3)	135 (31.5)	6.98 (5.23–9.3)	< 0.0001
*Taq*I	N = 253	N = 214		
Co-dominant				
TT	96 (38)	156 (73)	1.00 (Reference)^a^	
TC	96 (38)	36 (17)	4.3 (2.73–6.86)	< 0.0001
CC	61 (24)	22 (10)	4.51 (2.60–7.81)	< 0.0001
Dominant				
TT	96 (38)	156 (73)	1.00 (Reference)^a^	
TC/CC (TC + CC)	157 (76)	58 (27)	4.39 (2.96–6.52)	< 0.0001
Alleles	N = 506	N = 428		
T	288 (56.9)	348 (81.3)	1.00 (Reference)^a^	
C	218 (43.1)	80 (18.9)	3.29 (2.44–4.45)	< 0.0001

*N number of subjects, **OR Odds ratio, *95% CI* Confidence interval, ***P-values were considered significant when < 0.05. ^a^For details please see the Methods section

**Fig. 2:**
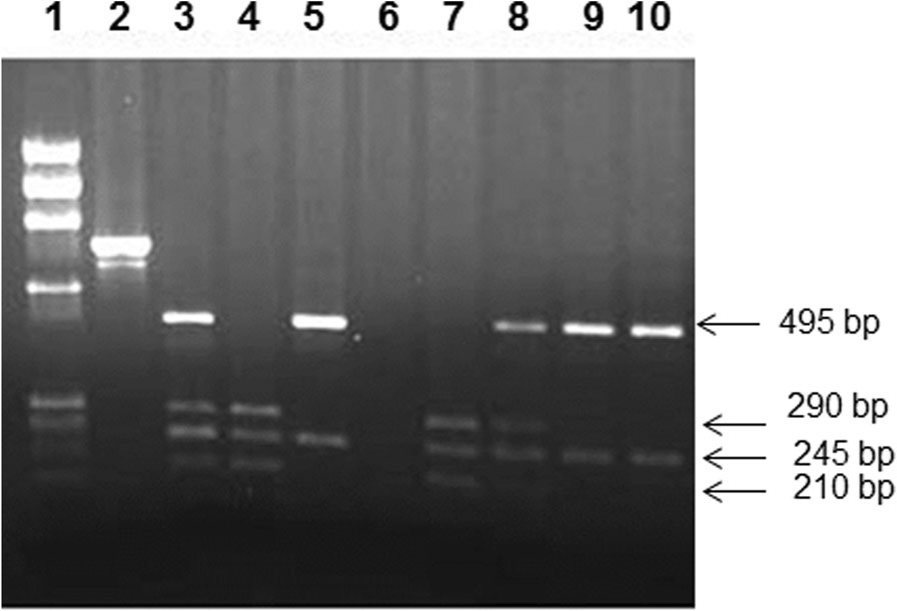
Detection of *VDR* gene *Taq*I polymorphism. PCR amplification of the genomic DNA was carried out and the products of amplification were cleaved with restriction enzyme *Taq*I (details given in Methods). Lane 1, phiX174 *Hae*III cut *M*_*r*_ markers; lane 2, uncleaved PCR product, lanes 3,8 *Taq*I cleavage pattern from subjects with heterozygous TC genotype; lanes 4,7 *Taq*I cleaved PCR product from subjects with CC genotype; lanes 5,9–10 *Taq*I cleaved PCR products from a subject with TT genotype, lane 6, no sample. The products were analyzed on 2% agarose gel and visualized under UV light after staining with ethidium bromide. The number on the right side indicate the product sizes (bp)

The method used for determining the genotypes of *VDR* gene *Taq*I polymorphism has been presented in Fig. 2. The frequency of homozygous variant ‘CC’ genotype was significantly higher in T1DM patients than in the controls (OR, 4.51 and 4.39 in co-dominant and dominant models respectively, Table 1). The same trend was manifested in the case of ‘C’ allele frequency (OR, 3.29, Table 1).

**Fig. 3:**
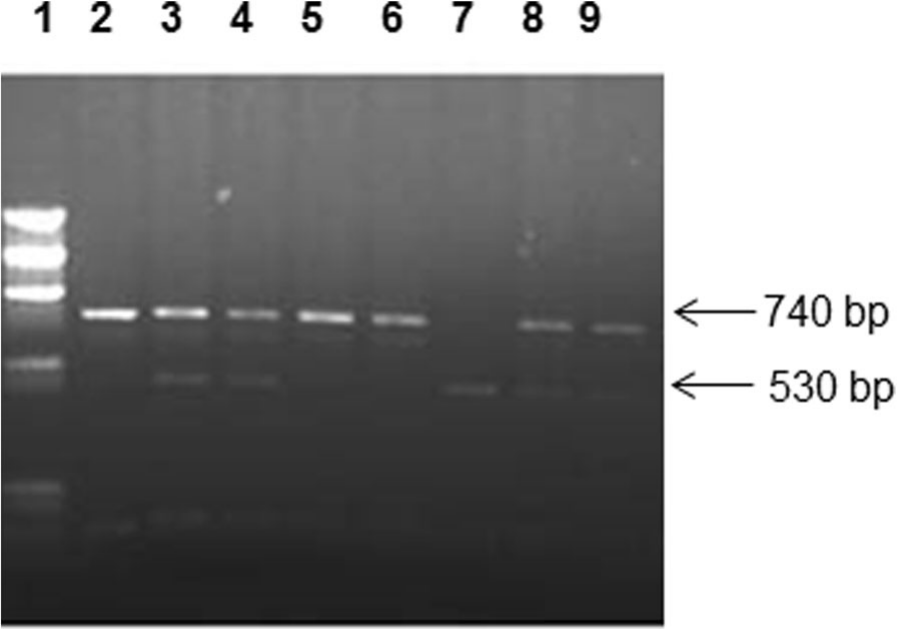
Detection of *VDR* gene *Apa*I polymorphism. PCR amplification of the genomic DNA was carried out and products of amplification were cleaved with restriction enzyme *Apa*I (details given in Methods). Lane 1, phiX174 *Hae*III cut *M*_*r*_ markers; lane 2, uncleaved PCR product; lanes 3,4,8,9, products from subjects with heterozygous GT genotype; lanes 5–6, products from subjects with homozygous TT genotype; lane 7, products from homozygous GG genotype. The numbers on the side are sizes (bp) of the characteristic bands resulting from PCR-RFLP analysis. The cleavage products were analyzed on 2% agarose gel and visualized under UV light after staining with Ethidium bromide

An example of the method used to determine genotypes of *VDR* gene *Apa*I polymorphism in Kuwaiti T1DM patients with different genotypes has been presented in Fig. 3. There was no significant difference between the frequencies of any of the genotype or allele between T1DM patients and the controls (Table 2). Although *Apa*I polymorphism was not informative in our cohort but like in many other populations, the ‘G’ allele was the ‘minor allele’ amongst Kuwaiti Arabs.

**Table 2:** Comparison of the genotype and allele frequency of *VDR* gene polymorphisms (*Apa*I*, and Bsm*I) between Kuwaiti T1DM patients and controls

*VDR* gene polymorphism	Patients *N* (%)	Control N* (%)	OR (95% CI)**	*P*–value***
*Apa*I	*N* = 252^a^	*N* = 214		
Co-dominant				
TT	192 (76.2)	162 (75.7)	1.00 (Reference)	
GT	31 (12.3)	37 (17.3)	0.71 (0.42–1.19)	0.23
GG	29 (11.5)	15 (7.0)	1.63 ((0.85–3.15)	0.15
Dominant				
TT	192 (76.2)	162 (75.7)	1.00 (Reference)	
GT/TT (GT + TT)	60 (23.8)	52 (24.3)	0.97 (0.64–1.49)	0.99
Alleles	*N* = 504	*N* = 428		
T	415 (82.3)	361 (84.3)	1.00 (Reference)	
G	89 (17.7)	67 (15.7)	1.16 (0.82–1.64)	0.47
*Bsm*I				
Co-dominant	*N* = 253	N = 214		
BB	141 (55.7)	120 (56.1)	1.00 (Reference)	
Bb	83 (32.8)	66 (30.8)	1.07 (0.71–1.60)	0.82
bb	29 (11.5)	28 (13.1)	0.88 (0.50–1.56)	0.78
Dominant				
BB	141 (55.7)	120 (56.1)	1.00 (Reference)	
Bb/bb (Bb + bb)	112 (44.3)	94 (43.9)	1.01 (0.70–1.46)	0.94
Alleles	N = 506	N = 428		
B	365 (72.1)	306 (71.5)	1.00 (Reference)	
b	141 (27.9)	122 (28.5)	0.97 (0.73–1.29)	0.88

*N number of subjects, **OR Odds ratio, *95% CI* Confidence interval; ***P-values were considered significant when < 0.05. ^a^Genotype could not be determined in one T1DM patient

**Fig. 4:**
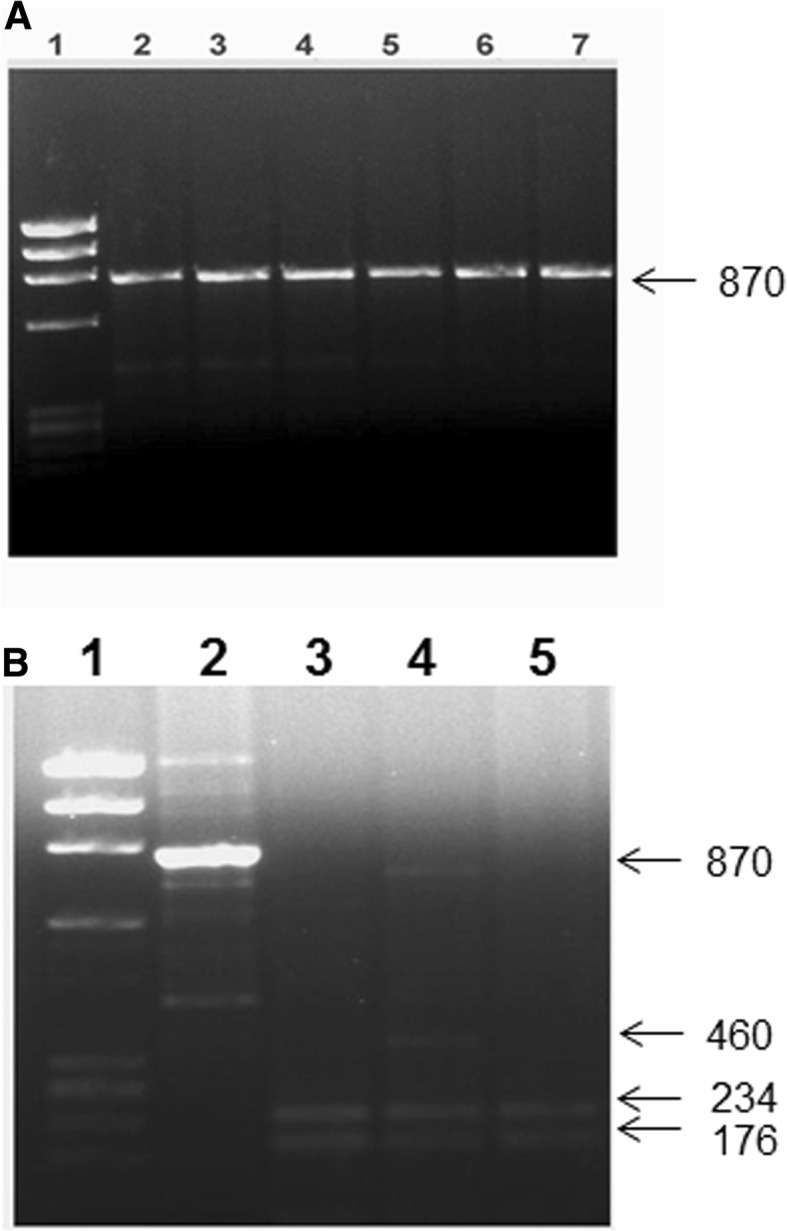
Detection of *VDR* gene *Bsm*I polymorphism. PCR amplification of genomic DNA was carried out and the products of amplification were cleaved with restriction enzyme *Mva*I (details given in Methods). A: lane 1, phiX174 *Hae*III cut *M*_*r*_ markers; lane 2, uncleaved PCR product; lanes 3–7, cleavage products from subjects with BB genotype. B: lane 1, ϕX174 *Hae*III cut *M*_*r*_ markers; lane 2, uncleaved PCR product; lane 3,5, cleavage products from subjects with bb genotype; lanes 4, cleavage products from subjects having Bb genotype. The numbers on the side are sizes (bp) of the characteristic bands resulting from PCR-RFLP analysis of the subjects having different genotypes. The restriction enzyme cleavage products were analyzed on 2% agarose gel and visualized under UV light after staining with Ethidium bromide

The genotype detection method used for *VDR* gene *Bsm*I polymorphism has been presented in Fig. 4. No significant difference was detected in any of the three genotypes (bb, Bb and BB) or the two alleles between T1DM patients and the controls (Table 2). The data on genotype and allele frequency showed that minor allele in the cohort was ‘b’ but it did not show any significant difference between patient and the control groups suggesting that this polymorphism was not associated with T1DM in the Kuwaiti cohort studied.

A comparison of the frequency of *VDR* gene *Fok*I polymorphism genotypes between different age-of-onset subgroups of T1DM patients has been presented in Table 3. In Kuwaiti T1DM patients with *Fok*I polymorphism genotypes ff and Ff (having at least one variant allele), statistically significant difference was detected between patients with age-of-onset < 4y and those with >6y and between age-of-onset subgroups 4-6y and > 6y respectively (Table 3). In T1DM patients with *Apa*I genotypes GG and GT (having at least one variant allele), statistically significant difference was detected between patients with age-of-onset < 4y and those with >6y and between subgroups 4-6y and > 6y (Table 4). In T1DM patients who had CC genotype for *Taq*I polymorphism showed statistically significant difference between < 4y and > 6y subgroups and none of the other subgroups (Table 5). However, the heterozygous CT genotype showed statistically significant difference between patients with age-of-onset < 4y and those with >6y and between subgroups 4-6y and > 6y (Table 5). In T1DM patients with *Bsm*I genotype bb, no significant difference was detected between any of the subgroups and for heterozygous Bb statistically significant difference was detected between patients with age-of-onset < 4y and those with >6y and between subgroups 4-6y and > 6y respectively (Table 6). Even in the case of homozygous ‘common’ genotypes (FF, TT, TT and BB), of the *VDR* gene polymorphisms, significant differences were noted between some age-of-onset subgroups (Tables 3, 4, 5, and 6).

**Table 3:** Comparison of the genotype frequency of *VDR* gene *Fok*I polymorphism among different age-of-onset subgroups of Kuwaiti T1DM patients

*FokI* Genotype/Age of onset sub-groups	Frequency (%)	OR (95% CI)*	*P*-value**
ff-genotype			
< 4 years vs	33/174 (19.0)	0.74 (0.44–1.23)	0.30
4–6 years	42/174 (24.1)		
< 4 years vs	33/174 (19.0)	0.18 (0.11–0.29)	< 0.0001
> 6 years	99/174 (56.9)		
4–6 years vs	42/174 (24.1)	0.24 (0.15–0.38)	< 0.0001
> 6 years	99/174 (56.9)		
Ff-genotype			
< 4 years vs	7/30 (23.3)	1.0 (0.30–3.30)	1
4–6 years	7/30 (23.3)		
< 4 years vs	7/30 (23.3)	0.27 (0.09–0.80)	0.03
> 6 years	16/30 (53.4)		
4–6 years vs	7/30 (23.3)	0.27 (0.09–0.80)	0.03
> 6 years	16/30 (53.4)		
FF-genotype			
< 4 years vs	8/44 (18.2)	0.48 (0.18–1.29)	0.21
4–6 years	14/44 (31.8)		
< 4 years vs	8/44 (18.2)	0.22 (0.08–0.58)	< 0.01
> 6 years	22/44 (50.0)		
4–6 years vs	14/44 (31.8)	0.47 (0.20–1.11)	0.13
> 6 years	22/44 (50.0)		

*OR (95% CI), odds ratio 95% confidence interval; **P-values were considered significant when < 0.05. The age-of-onset information was not available in 5 T1DM patients (data reported from 248 T1DM patients)

**Table 4:** Comparison of the frequency of *VDR* gene *Apa*l polymorphism genotypes among different age-of-onset sub-groups of Kuwaiti TIDM patients

*ApaI* Genotype/Age of onset sub-groups	Frequency (%)	OR (95% CI)*	*P*-value**
GG-genotype			
< 4 years vs	7/28 (25)	1.53 (0.42–5.57)	0.74
4–6 years	5/28 (18)		
< 4 years vs	7/28 (25)	0.25 (0.08–0.77)	0.02
> 6 years	16/28 (57)		
4–6 years vs	5/28 (18)	0.16 (0.04–0.55)	0.005
> 6 years	16/28 (57)		
GT-genotype			
< 4 years vs	7/31 (22.6)	1.22 (0.36–4.14)	1.0
4–6 years	6/31 (19.4)		
< 4 years vs	7/31 (22.6)	0.21 (0.07–0.64)	< 0.01
> 6 years	18/31 (58.0)		
4–6 years vs	6/31 (19.4)	0.17 (0.06–0.54)	< 0.01
> 6 years	18/31 (58.0)		
TT-genotype			
< 4 years vs	34/188 (18.0)	0.58 (0.35–0.94)	0.04
4–6 years	52/188 (27.7)		
< 4 years vs	34/188 (18.0)	0.19 (0.12–0.30)	< 0.0001
> 6 years	102/188 (54.3)		
4–6 years vs	52/188 (27.7)	0.32 (0.21–0.50)	< 0.0001
> 6 years	102/188 (54.3)		

*OR (95% CI), odds ratio 95% confidence interval; **P-values were considered significant when < 0.05. The age-of-onset information was not available in 5 T1DM patients (data available from 248 T1DM patients). In one T1DM patient the *Apa*I genotype could not be determined, therefore data reported from 247 cases

**Table 5:** Comparison of the frequency of *VDR* gene *TaqI* polymorphism genotypes among different age-of-onset sub-groups of Kuwaiti TIDM patients

*Taq*I genotype/Age of onset sub-groups	Frequency (%)	OR (95% CI)*	*P*-value**
CC-genotype			
< 4 years vs	11/60 (18.3)	0.39 (0.17–0.90)	0.04
4–6 years	22/60 (36.7)		
< 4 years vs	11/60 (18.3)	0.27 (0.12–0.63)	< 0.01
> 6 years	27/60 (45.0)		
4–6 years vs	22/60 (36.7)	0.71 (0.34–1.47)	0.46
> 6 years	27/60 (45.0)		
CT-genotype			
< 4 years vs	22/96 (22.9)	1.29 (0.64–2.59)	0.59
4–6 years	18/96 (18.8)		
< 4 years vs	22/96 (24.5)	0.21 (0.11–0.40)	< 0.0001
> 6 years	56/96 (58.3)		
4–6 years vs	18/96 (18.8)	0.16 (0.09–0.32)	< 0.0001
> 6 years	56/96 (58.3)		
TT -genotype			
< 4 years vs	15/92 (16.3)	0.58 (0.28–1.20)	0.20
4–6 years	23/92 (25.0)		
< 4 years vs	15/92 (16.3)	0.14 (0.07–0.27)	< 0.0001
> 6 years	54/92 (58.7)		
4–6 years vs	23/92 (25.0)	0.23 (0.13–0.44)	< 0.0001
> 6 years	54/92 (58.7)		

*OR (95% CI), odds ratio 95% confidence interval; **P-values were considered significant when < 0.05. The age-of-onset information was not available in 5 T1DM patients (data reported from 248 T1DM patients)

**Table 6:** Comparison of the frequency of *VDR* gene *Bsm*I polymorphism genotypes among different age-of-onset sub-groups of Kuwaiti TIDM patients

*Bsm*I genotype/Age of onset sub-groups	Frequency (%)	OR (95% CI)*	*P*-value**
bb - genotype			
< 4 years vs	7/29 (24.1)	0.84 (0.26–2.71)	1.0
4–6 years	8/29 (27.6)		
< 4 years vs	7/29 (24.1)	0.34 (0.11–1.05)	0.10
> 6 years	14/29 (48.3)		
4–6 years vs	8/29 (27.6)	0.41 (0.14–1.22)	0.18
> 6 years	14/29 (48.3)		
Bb-genotype			
< 4 years vs	17/83 (20.5)	0.87 (0.41–1.82)	0.85
4–6 years	19/83 (22.9)		
< 4 years vs	17/83 (20.5)	0.20 (0.10–0.39)	< 0.0001
> 6 years	47/83 (56.6)		
4–6 years vs	19/83 (22.9)	0.23 (0.12–0.45)	< 0.0001
> 6 years	47/83 (56.6)		
BB-genotype			
< 4 years vs	24/136 (17.6)	0.60 (0.33–1.07)	0.11
4–6 years	36/136 (26.5)		
< 4 years vs	24/136 (17.6)	0.17 (0.20–0.30)	< 0.0001
> 6 years	76/136 (55.9)		
4–6 years vs	36/136 (26.5)	0.28 (0.17–0.47)	< 0.0001
> 6 years	76/136 (55.9)		

*OR (95% CI), odds ratio 95% confidence interval; **P-values were considered significant when < 0.05. The age-of-onset information was not available in 5 T1DM patients (data reported from 248 T1DM patients)

The allele frequencies from four *VDR* gene polymorphisms were used to build haplotypes as described earlier (18). Based on this scheme, fifteen haplotypes were constructed from the data obtained in Kuwaiti T1DM patients and controls. The frequency of each of these haplotype from T1DM patients and controls has been presented in Table 7. Of the total fifteen haplotypes, significant differences were detected between the controls and T1DM patients in seven haplotypes (Table 7). From these seven haplotypes, the highest OR were detected in two haplotypes, [f/C/T/b] *P* < 0.002 and [f/C/T/B] *P* < 0.0001 (Table 7). The frequency of both these haplotypes was considerably higher in T1DM patients than in the controls and collectively, these two haplotypes were detected in nearly 34% of all the Kuwaiti T1DM patients. This further highlighted the positive association of variant alleles (f) of the *VDR* gene *Fok*I and *Taq*I polymorphisms with genetic predisposition of T1DM in Kuwaiti Arab children.

**Table 7:** Comparison of *VDR* gene haplotype frequencies in Kuwaiti T1DM patients and controls

Haplotype^a^	Patients (%) *N* = 202	Controls (%) *N* = 154	Odds ratio (95% CI)*	*P*-value**
f/C/G/b	6 (3)	0	10.22 (0.571–182.97)	0.038
f/C/G/B	5 (2.5)	1 (0.6)	3.88 (0.448–33.603)	0.24
F/C/G/B	1 (0.5)	2 (1.3)	0.38 (0.034–4.211)	0.58
f/T/G/b	5 (2.5)	6 (3.9)	0.63 (0.187–2.091)	0.54
F/T/G/b	0	2 (1.3)	0.15 (0.007–3.163)	0.18
f/T/GB	6 (3)	16 (10.4)	0.26 (0.100–0.69)	0.006
F/T/G/B	2 (1)	7 (4.5)	0.21 (0.043–1.03)	0.04
*f/C/T/b*	**23 (11.4)**	**4 (2.6)**	**4.81 (1.630–14.245)**	**0.002** ^b^
F/C/T/b	8 (4)	10 (6.5)	0.59 (0.228–1.543)	0.33
*f/C/T/B*	**45 (22.3)**	**6 (3.9)**	**7.07 (2.929–17.065)**	**< 0.0001** ^b^
F/C/T/B	14 (6.9)	14 (9.1)	0.75 (0.345–1.613)	0.58
f/T/T/b	20 (9.9)	14 (9.1)	1.10 (0.536–2.253)	0.93
F/T/T/b	6 (3)	20 (13)	0.21 (0.080–0.524)	0.0004
f/T/T/B	48 (23.8)	25 (16.2)	1.61 (0.939–2.752)	0.11
F/T/T/B	13 (6.4)	27 (17.5)	0.32 (0.161–0.651)	0.002

^a^The *VDR* gene haplotypes were documented as described earlier [18] and the haplotypes were described in the order *Fok*I/*Taq*I/*Apa*I/*Bsm*I; ^b^ The haplotypes containing majority of variant alleles and with highest OR are shown in bold. *OR (95% CI), Odds ratio at 95% Confidence interval; * *P*-values were considered significant when < 0.05

The frequency of positivity for three autoantibodies (ICA, GADA and INS) was determined in T1DM patients with different genotypes of four *VDR* gene polymorphisms and has been presented in Table 8. In the case of ICA, the frequency of autoantibody-positivity was detected in approximately 48–66% of the T1DM patients (Table 8). The GADA-positivity was found in nearly 75–89% T1DM patients carrying different *VDR* gene polymorphism genotypes (Table 8). The INS-positivity was observed in approximately 65–79% of the T1DM patients having different *VDR* gene polymorphism genotypes (Table 8). Nearly all the T1DM patients harbored multiple autoantibodies (data not shown).

**Table 8:** Association of *VDR* gene polymorphisms (*Fok*I*, Taq*I*, Bsm*I *and Apa*I*)* genotypes with three autoantibodies, ICA (islet cell autoantibody), GADA (glutamic acid decarboxylase) and INS (insulin autoantibody) in Kuwaiti T1DM patients

Genotype	ICA + ve* (%)	GADA + ve* (%)	INS + ve* (%)
*Fok*I			
ff	101/178 (57)	146/178 (82)	126/178 (71)
Ff	16/30 (53)	26/30 (87)	21/30 (70)
FF	25/45 (57)	37/45 (82)	30/45 (68)
*Taq*I			
CC	35/61 (57)	52/61 (85)	43/61 (71)
TC	53/96 (55)	85/96 (89)	70/96 (73)
TT	54/96 (56)	72/96 (75)	64/96 (67)
*BsmI*			
bb	19/29 (66)	24/29 (83)	23/29 (79)
Bb	46/83 (55)	72/83 (87)	54/83 (65)
BB	77/141 (55)	113/141 (80)	100/141 (71)
*Apa*I**			
GG	14/29 (48)	23/29 (79)	21/29 (72)
TG	19/31 (61)	26/31 (84)	23/31 (74)
TT	109/192 (57)	159/192 (83)	133/192 (69)

*The positivity of autoantibodies was expressed as percentage of subjects carrying a specific autoantibody in comparison to the total number with a particular *VDR* genotype. Each patient carried multiple autoantibodies. **In one T1DM patient, the genotype for *Apa*I, could not be determined

## Discussion

This study investigated an association of four *VDR* gene polymorphisms with susceptibility of T1DM in Kuwaiti Arabs by comparing the genotype frequencies between patients and the controls. A statistically significant association was detected between *VDR* gene *Fok*I polymorphism and T1DM in Kuwaiti Arab children (Table 1). The *Fok*I ‘f’ polymorphism results from a C➔T transition at the junction of intron 1 and exon 2 and creates an additional initiation codon (ATG) located three codons downstream to the original start site [43]. The shorter VDR protein (424 aa, which results from FF genotype) is more active than the longer form (427 aa produced from ff-genotype [46]. Previous studies have demonstrated that the proportion of CD4^+^ cells was considerably lower in Th-cells (under vitamin D stimulation) from T1DM patients carrying the FF genotype compared to patients with Ff or ff genotypes [47]. It has been postulated that since the f-allele corresponds to a less active VDR protein [48, 49], the ff-genotype can possibly contribute to the development of T1DM either by causing weaker insulin production or by affecting vitamin D’s immunosuppressive properties [50, 51]. A study from Brazil has reported that individuals with ff and Ff genotypes tended to have lower residual pancreatic beta-cell function [52]. Findings similar to our results from Kuwaiti Arabs have been reported from Egypt, where a significantly higher frequency of genotype Ff of the *Fok*I polymorphism was found in T1DM patients compared to the controls [53]. However, in contrast to the Egyptian data, where the *Fok*I and *Bsm*I polymorphisms were correlated with T1DM, our result did not find any significant association between *Bsm*I polymorphism and the T1DM in Kuwaiti Arab children. The results on haplotypes constructed from the genotype frequencies observed in Kuwaiti T1DM patients (Table 7) further support a significant correlation of the *VDR* gene *Fok*I and *Taq*I polymorphisms with susceptibility to T1DM.

In contrast to the *Fok*I polymorphism which has functional implications, the *Apa*I, *Taq*I and *Bsm*I polymorphisms are located near 3’end of the *VDR* gene. Our results from Kuwaiti Arab children showed a positive association between the C-allele of *VDR* gene *Taq*I polymorphism and T1DM while no association was found between T1DM and the *Apa*I and *Bsm*I polymorphisms. Similar results have been reported from Korean T1DM patients, in which TT genotype of the *Taq*I polymorphism was shown to have decreased risk compared to the homozygous CC and heterozygous TC genotypes [32]. In contrast to the positive associations with *Taq*I C-allele from Kuwait and Korea, the results reported from Germany, showed that frequency of TT genotype of *VDR* gene *Taq*I polymorphism was higher in T1DM patients than in the controls [54]. The three *VDR* gene polymorphisms, *Apa*I, *Taq*I and *Bsm*I have been shown to be in strong linkage disequilibrium with each other in the Caucasians but no significant linkage disequilibrium was reported with the *Fok*I polymorphism [55]. The data reported here from Kuwaiti Arabs is markedly different from that in the Caucasians because the association with T1DM was detected only in the case of *Taq*I polymorphism. In a meta-analysis involving Chinese adult samples, it was concluded that possession of *VDR* gene *Bsm*I polymorphism would increase the risk of T1DM in Asians, South Americans, Africans and Turks [37]. As stated earlier, the three polymorphisms i.e. *Apa*I, *Taq*I and *Bsm*I are located at the 3′ untranslated region (3’UTR) of the *VDR* gene [48]. The 3’UTR is known to be involved in regulation of gene expression, possibly through the control of mRNA stability [44, 56, 57]. Our results from Kuwaiti Arab children and those reported from other populations (mentioned above), highlight the presence of a population-specific pattern of T1DM susceptibility which most likely result from an interaction of diverse and unique genetic factors with the environmental determinants that might exist in different geographical regions.

The pattern of association between *VDR* gene polymorphism genotypes and age-of-onset subgroups of Kuwaiti T1DM patients showed almost a similar pattern except for the *Bsm*I polymorphism (Tables 3, 4, 5 and 6), mostly significant differences were detected between subgroups < 4y and > 6y and between 4-6y and > 6y subgroups. This is in contrast to the data reported from Brazil in which no association was found between the onset of T1DM and age at diagnosis [58]. A study from Japan showed a significantly high association between *VDR* gene *Bsm*I polymorphism allele B and the acute-onset type 1 diabetes [59]. It has been suggested that the variability in association of the *VDR* gene polymorphisms with T1DM in different populations may possibly be due to differences in their ethnic background, diverse evolutionary lineages, and interactions with other genetic and/or environmental factors which influence the pathogenesis of T1DM [24, 55].

The pattern of autoantibody-positivity was also analyzed in Kuwaiti T1DM patients harboring different genotypes of four *VDR* gene polymorphisms (Table 8). A similar distribution pattern (50–85% positivity) was observed amongst different *VDR* genotypes with the highest positive correlation in the case of GADA autoantibody. This is markedly different from a previous study from Japan in which no association was found between GADA and IA-2 autoantibodies and the acute-onset type 1 diabetes [31, 59]. Several previous reports have described the autoantibodies to pancreatic antigens as a characteristic feature of T1DM [59]. The autoantibody association data with T1DM from India showed a relatively lower positive correlation between T1DM and three autoantibodies (GADA, ZnT8 and IA-2) [60]. Only 47% of the Indian T1DM patients carried one or more of these autoantibodies and highest positive correlation was seen with GADA (detected in 38% T1DM patients) [60]. In reports from Western populations, 85–90% of the newly diagnosed T1DM patients were shown to carry either GADA, IA-2, ZnT8 or INS autoantibody [61–64]. A higher incidence of GADA-positivity (73%), followed by ICA-2 (42%) has been reported from Saudi Arabia [65]. In contrast, GADA-positivity was reported in only 31% of the T1DM patients from China [66]. In comparison to previous reports from Asia, our findings from Kuwaiti T1DM patients showed a relatively higher positivity rate of GADA and INS autoantibodies. The GADA autoantibodies were detected in > 80% and the INS autoantibodies in > 70% Kuwaiti T1DM patients (Table 8). The autoantibody positivity in Kuwaiti T1DM patients who carried the variant genotypes of *VDR* gene *Fok*I and *Taq*I polymorphisms was slightly higher than that of the non-variant genotypes. Another significant feature of our results from Kuwaiti T1DM patients was that multiple autoantibodies were detected in each patient. The results reported here on *VDR* gene *Fok*I and *Taq*I polymorphisms and autoantibodies along with those reported earlier [39] contribute significantly in identifying the determinants of genetic predisposition to T1DM in Kuwaiti Arab children.

## Conclusions

The findings reported in this study show that the *VDR* gene *Fok*I and *Taq*I polymorphisms are associated with susceptibility to T1DM in Kuwaiti Arabs. The *VDR* gene polymorphisms *Apa*I and *Bsm*I did not show a positive association with T1DM. However, the frequency of all four *VDR* gene polymorphisms was higher in the late age-of-onset T1DM patients compared to the early age-of-onset cases from Kuwait. Three autoantibodies (ICA, GADA and INS) showed varying degree of positive association in T1DM patients carrying different genotypes of four *VDR* gene polymorphisms with the highest positivity observed in the case of GADA autoantibody. The *VDR* gene polymorphisms (*Fok*I and *Taq*I) and three autoantibodies contribute significantly to the genetic predisposition of T1DM Kuwaiti Arabs.

## Abbreviations

bp: Base pair
CD4 +: Cluster of differentiation 4
CI: Confidence interval
EDTA: Ethylene diamine tetra acetic acid
GADA: Glutamic acid decarboxylase 65KDa autoantibody
HbA1c: Glycated hemoglobin
HPLC: High performance liquid chromatography
ICA: Islet-cell autoantibody
INS: Insulin autoantibody
ISPAD: International Society of Pediatric and Adolescent Diabetes
NOD: Non-obese diabetic mouse
OR: Odds ratio
PCR-RFLP: Polymerase chain reaction-restriction fragment length polymorphism
SNP: Single nucleotide polymorphism
SPSS: Statistical package for social sciences, *M*_*r*_, molecular size marker
T1DM: Type 1 diabetes mellitus
Th2: T helper cell type 2
UV: Ultraviolet light
VDR: Vitamin D receptor
